# Health promotion among unemployed people – meta-analytic evidence

**DOI:** 10.1093/eurpub/ckac131.316

**Published:** 2022-10-25

**Authors:** A Hollederer, K Paul

**Affiliations:** 1 Faculty of Human Sciences, University Kassel, Kassel, Germany; 2 Faculty of Business, Economics and Law, Friedrich-Alexander-University of Erlangen-Nü, Nuremberg, Germany

## Abstract

**Background:**

It is well known that unemployment impairs health. However, less is known about health promotion among unemployed people and healthy coping strategies to deal with unemployment.

**Methods:**

A random-effect meta-analysis was applied to statistically integrate quantitative controlled intervention studies among unemployed people.

**Results:**

The meta-analysis included 34 eligible primary studies with 36 independent intervention samples. For mental health, the average meta-analytic effect sizes for the comparison of intervention group and comparison group was significant and of small size after the intervention d = 0.25; 95% CI [0.10, 0.39], and at follow-up, d = 0.13; 95% CI [0.04, 0.22]. For health behavior (measured as physical activity), the effects were also significant after the intervention, d = 0.30; 95% CI [0.12, 0.47] (no data available at follow-up). Effects on self-assessed physical health status were very small and not significant. However, when health improvement was the only goal of the respective intervention (in contrast to studies with multiple goals, e.g., health improvement plus re-employment), the effect on physical health was significant, too, d = 0.15; 95% CI [0.04, 0.25]. Moderator tests showed that intervention effects on mental health were significantly stronger when cognitive-behavioral methods were used (p < .05), while the use other kinds of stress-management techniques (p < .05), of methods to increase social support (p < .05), or of relaxation techniques (p < .07) did not improve the effectiveness of the intervention.

**Conclusions:**

The present meta-analyses provided evidence that, on average, interventions aiming at improving unemployed people’s health do indeed improve participants’ health.

**Key messages:**

• The present meta-analysis confirms that it is possible to promote unemployed people’ health. The moderator effects for type of intervention that were found here might be helpful for health promotion.

• The inclusion of cognitive-behavioral methods, appears to be a successful approach for health-oriented interventions with unemployed people.

